# RNA Silencing of Mcl-1 Enhances ABT-737-Mediated Apoptosis in Melanoma: Role for a Caspase-8-Dependent Pathway

**DOI:** 10.1371/journal.pone.0006651

**Published:** 2009-08-17

**Authors:** Angela M. Keuling, Kathleen E. A. Felton, Arabesque A. M. Parker, Majid Akbari, Susan E. Andrew, Victor A. Tron

**Affiliations:** 1 Department of Medical Genetics, University of Alberta, Edmonton, Alberta, Canada; 2 Department of Pathology and Molecular Medicine, Queen's University, Kingston, Ontario, Canada; 3 Department of Pathology, Vancouver Coastal Health, Lions Gate Hospital Site, Vancouver, British Columbia, Canada; Roswell Park Cancer Institute, United States of America

## Abstract

**Background:**

Malignant melanoma is resistant to almost all conventional forms of chemotherapy. Recent evidence suggests that anti-apoptotic proteins of the Bcl-2 family are overexpressed in melanoma and may contribute to melanoma's striking resistance to apoptosis. ABT-737, a small-molecule inhibitor of Bcl-2, Bcl-xl and Bcl-w, has demonstrated efficacy in several forms of leukemia, lymphoma as well as solid tumors. However, overexpression of Mcl-1, a frequent observance in melanoma, is known to confer ABT-737 resistance.

**Methodology/Principal Findings:**

Here we report that knockdown of Mcl-1 greatly reduces cell viability in combination with ABT-737 in six different melanoma cell lines. We demonstrate that the cytotoxic effect of this combination treatment is due to apoptotic cell death involving not only caspase-9 activation but also activation of caspase-8, caspase-10 and Bid, which are normally associated with the extrinsic pathway of apoptosis. Caspase-8 (and caspase-10) activation is abrogated by inhibition of caspase-9 but not by inhibitors of the death receptor pathways. Furthermore, while caspase-8/-10 activity is required for the full induction of cell death with treatment, the death receptor pathways are not. Finally, we demonstrate that basal levels of caspase-8 and Bid correlate with treatment sensitivity.

**Conclusions/Significance:**

Our findings suggest that the combination of ABT-737 and Mcl-1 knockdown represents a promising, new treatment strategy for malignant melanoma. We also report a death receptor-independent role for extrinsic pathway proteins in treatment response and suggest that caspase-8 and Bid may represent potential markers of treatment sensitivity.

## Introduction

Over the past 40 years, the incidence of melanoma has increased more rapidly than any other type of cancer [1]. If melanoma is diagnosed early, it can be cured by surgical removal of the tumor [2]. However, metastatic melanoma is usually incurable, with a 5-year survival rate less than 10% and a median survival time of 7.5 months after diagnosis [3]. Currently, dacarbazine (DTIC) is the standard treatment for advanced cases of melanoma; however, complete remission is achieved in only 5% of patients [4]. In the past few years, new treatments have been developed but, as yet, none have significantly prolonged survival time [4], [5], [6]. Recent studies have suggested that the Bcl-2 family of apoptotic proteins plays a critical role in chemoresistance in melanoma [7].

The Bcl-2 family consists of both pro- and anti-apoptotic proteins. Pro-apoptotic Bcl-2 proteins are further divided into multidomain and BH3-only proteins. The multidomain pro-apoptotic proteins Bak and Bax oligomerize in the mitochondrial membrane to allow release of cytochrome c and other apoptotic effectors into the cytoplasm [8]. Bak and Bax activity are facilitated by BH3-only proteins (e.g. Bim, Bid, Bad, Noxa, and Puma) and inhibited by anti-apoptotic Bcl-2 proteins (Bcl-2, Bcl-xL, Mcl-1, Bcl-w and A1) [9], [10], [11], [12].

A number of studies have reported overexpression of Bcl-2, Mcl-1 and Bcl-xL in melanoma compared to normal tissue or benign nevi, although there is some controversy as to the role of Bcl-2 expression in chemoresistance [7], [13], [14], [15]. Therapeutic strategies to reduce levels of these proteins enhance the effects of conventional chemotherapeutics in pre-clinical melanoma models [reviewed in 5].

ABT-737 is a potent small-molecule inhibitor of Bcl-xL, Bcl-2 and Bcl-w (K_i_≤1 nM), which has demonstrated single-agent activity in a number of hematopoietic cancers and solid tumors in pre-clinical trials [16], [17], [18], [19]. However, several studies have shown that high levels of Mcl-1 confer ABT-737 resistance [16], [20], [21]. Concordantly, down-regulation of Mcl-1 by genetic and chemical strategies restores treatment sensitivity. The combination of Mcl-1 down-regulation and ABT-737 appears to be an efficient means of inducing apoptosis in multiple tumor types.

A recent study demonstrated that ABT-737 induces cell death in melanoma cell lines when combined with proteasome inhibitor MG-132 [22] The authors also perform an experiment indicating that ABT-737-dependent cell death can be enhanced by knockdown of Mcl-1. Here we confirm this observation and further provide the first in-depth characterization of the combined effect of Mcl-1 small interfering RNA (siRNA) and ABT-737 in malignant melanoma. We examined the effects of both single agents and the combination treatment on the induction of cell death in six melanoma cell lines. While neither single agent induces a significant amount of death in all cell lines, the combination treatment is consistently effective in reducing overall viability and inducing apoptosis in melanoma cell lines. Furthermore, we observed that the combination treatment was accompanied by death receptor-independent activation of caspase-8, caspase-10, and Bid. Finally, we demonstrate correlations between steady-state levels of cleaved caspase-8 and Bid and sensitivity to the combination treatment suggesting their potential as markers for measuring treatment efficacy. Overall, our studies demonstrate that the combination treatment of Mcl-1 knockdown and ABT-737 is a promising new treatment strategy for melanoma that calls for further investigation *in vivo*.

## Results

### Overexpression of Mcl-1 in Melanoma

**Figure 1:**
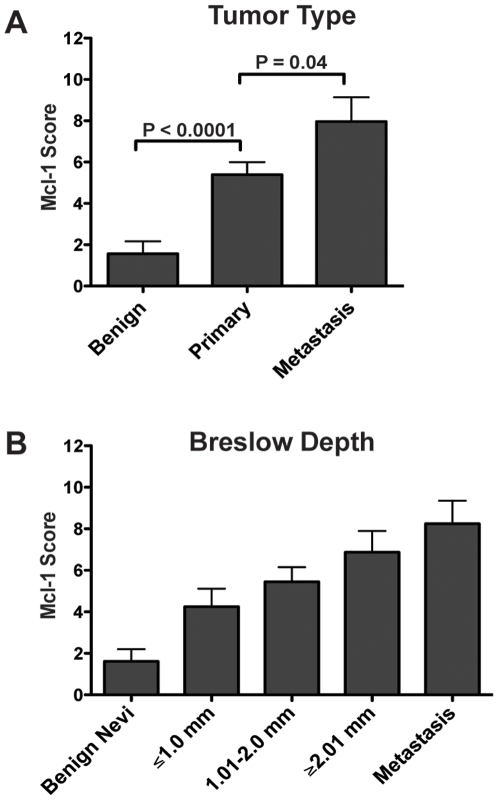
Results of tissue microarray demonstrating overexpression of Mcl-1 in melanoma. (A) Increased Mcl-1 staining in primary melanoma compared to benign nevi (p<0.0001) and in melanoma metastases compared to primary melanoma (p = 0.04). Data represent mean±SEM. *P* values are based on two-tailed, unpaired *t* tests. (B) Mcl-1 expression increases with tumor depth. Data represent mean±SEM.

In a previous study from our group, we reported increased expression of Mcl-1 in malignant melanoma compared to benign nevi in an immunohistochemical comparison of 5 nevi and 15 melanoma samples (10 primary and 5 metastases) [7]. In the current study, we expanded these results utilizing a tissue microarray. By comparing 25 benign nevi and 65 melanoma samples (41 primary and 24 metastases), we observed a statistically significant difference in Mcl-1 score between benign nevi and primary melanoma (P<0.0001) and between primary melanoma and metastatic disease (P = 0.04) (Figure 1A). Furthermore, there was a trend towards increased Mcl-1 levels with increased tumor depth (Figure 1B).

### Knockdown of Mcl-1 using Dicer-substrate siRNA

**Figure 2:**
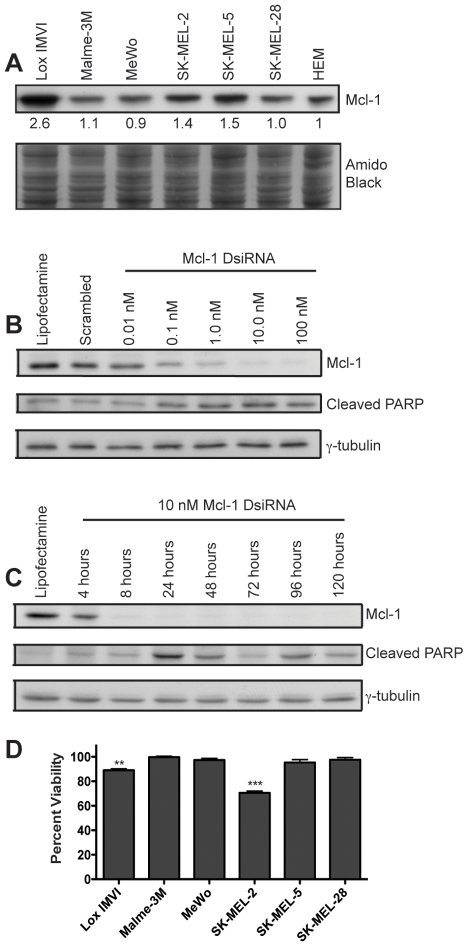
Knockdown of Mcl-1 with Dicer substrate siRNA (DsiRNA). (A) Immunoblot showing endogenous Mcl-1 levels in six melanoma cell lines compared to normal human epidermal melanocytes (HEM). Cells were harvested for protein at approximately equal confluency one day after splitting. Values represent quantification of bands using the amido black stain to correct for loading and normalization to the HEM band. (B) Western blot analysis demonstrating dose-dependent knockdown by Mcl-1 DsiRNA. Lox IMVI cells were treated with Lipofectamine-only, 100 nM Scrambled DsiRNA or increasing doses of Mcl-1 DsiRNA for 24 hours. Immunoblotting was performed using antibodies directed against Mcl-1, cleaved PARP (apoptosis marker) and γ–tubulin (loading control). (C) Western blot analysis showing time course of Mcl-1 knockdown. Lox IMVI cells were treated with Lipofectamine-only for 24 hours or with 10 nM Mcl-1 DsiRNA for the indicated time points. (D) Cell viability following Mcl-1 DsiRNA treatment. Cells were transfected with 10 nM Mcl-1 or Scrambled DsiRNA for 24 hours then assessed for viability by MTT assay. Viability is expressed as a percentage of the Scrambled treatment. Data represent mean±SEM (n = 4). Significant differences between the Mcl-1 and Scrambled treatments were determined using two-tailed, unpaired *t* tests. **P≤0.005, ***P≤0.0005.

To examine the effects of Mcl-1 knockdown and ABT-737 on melanoma *in vitro*, we used six melanoma cell lines: Lox IMVI, Malme-3M, MeWo, SK-MEL-2, SK-MEL-5 and SK-MEL-28. All cell lines, with the exception of MeWo, are part of the National Cancer Institute's NCI-60 panel of cancer cell lines. We observed detectable Mcl-1 expression in all cell lines, with three of the six displaying increased expression compared to normal human melanocytes (Figure 2A). To reduce Mcl-1 protein levels, we used Dicer-substrate siRNA (DsiRNA). These 27-mer RNA duplexes display an up to 100-fold increase in potency compared to conventional siRNA [23]. Indeed, we observed noticeably reduced levels of Mcl-1 with as little as 0.01 nM Mcl-1 DsiRNA. In all cell lines, maximum knockdown is achieved at a dose of 10 nM DsiRNA (Figure 2B and data not shown). When optimizing knockdown, we also examined levels of cleaved PARP as an indication of apoptosis induction. Increased PARP cleavage was not observed in all cell lines; however, in Lox IMVI, we observed maximum PARP cleavage at the 10 nM dose (Figure 2B). We therefore utilized 10 nM as a standard dose in subsequent assays. In all cell lines, maximum knockdown is achieved at 24 hours and is maintained for up to 5 days (Figure 2C and data not shown). We observed maximum PARP cleavage at 24 hours in Lox IMVI and thus 24 hours is used as our standard time point. We next determined whether Mcl-1 knockdown alone could reduce viability of melanoma cells. Compared to Scrambled DsiRNA, Mcl-1 DsiRNA significantly decreases viability in two cell lines, Lox IMVI and SK-MEL-2 (Figure 2D).

### Combination of Mcl-1 knockdown and ABT-737 decreases viability in multiple melanoma cell lines

**Figure 3:**
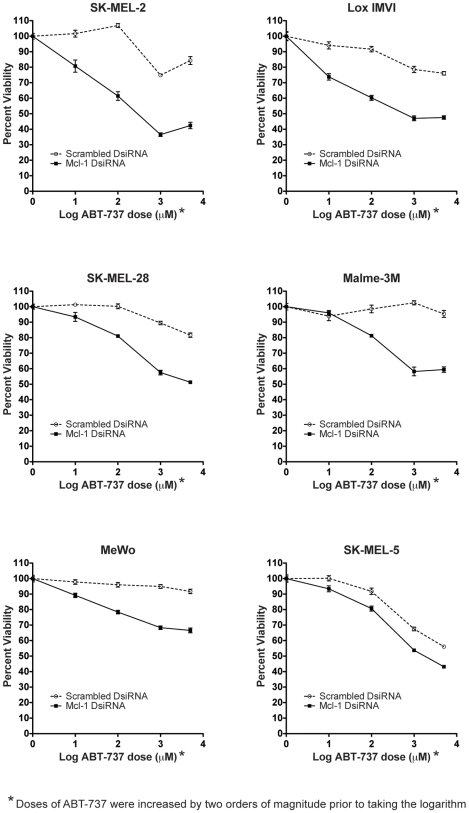
Synergistic effect of Mcl-1 knockdown and ABT-737 on cell viability in melanoma cell lines. Cells were transfected with either 10 nM Mcl-1 or Scrambled DsiRNA and treated with 0, 0.1, 1, 10, or 50 µM of ABT-737. Each dose was increased by two orders of magnitude before the logarithm was taken to allow us to graph all doses as positive values. Viability was assessed 24 hours post-transfection via MTT assay. Each curve was normalized back to the 0 µM ABT-737 treatment: the Scrambled DsiRNA curve was normalized to cells treated with only Scrambled DsiRNA (10 nM) and the Mcl-1 DsiRNA curve was normalized to cells treated with only Mcl-1 DsiRNA (10 nM). Data represent mean±SEM (n = 4).

As knockdown of Mcl-1 alone was not effective in the induction of cell death in all cell lines, we tested the effects of ABT-737, which inhibits Bcl-2, Bcl-xl and Bcl-w [17]. Bcl-2 and Bcl-xL are both known to be upregulated in melanoma [24]. ABT-737, alone or in combination with Scrambled DsiRNA, has only minor effects on cell viability in the six cell lines (Figure 3 and Supplementary Figure S1). SK-MEL-5 displays the strongest response; however, even at the highest dose of ABT-737, viability remains over 50%. Furthermore, Malme-3M and MeWo show little to no response. We therefore examined the combined effect of Mcl-1 knockdown and ABT-737.

In contrast to the single treatments, the combination of Mcl-1 DsiRNA and ABT-737 decreases viability in all cell lines examined (Figure 3). To assess the synergistic interaction between Mcl-1 knockdown and ABT-737, we used two-way ANOVA calculations as previously described [25]. In all cell lines there was a statistically significant interaction between the two treatments, with cell lines varying in the extent of the synergism. SK-MEL-5 displays the weakest effect of the interaction (i.e. interaction accounts for 1.97% of total variation, P = 0.001) whereas Malme-3M shows the greatest synergistic effect (interaction accounts for 24.62% of variation, P<0.0001) The Malme-3M cell line shows only minor effects of the single treatments but displays a strong viability decrease with the combination treatment, illustrating the strength of the synergistic effect of the combination therapy.

### Mcl-1 knockdown and ABT-737 induce apoptotic cell death

**Figure 4:**
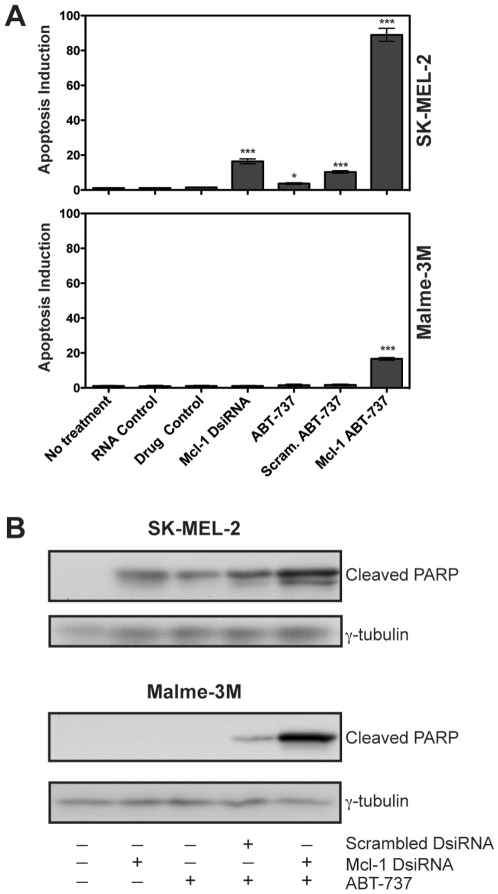
Mcl-1 knockdown and ABT-737 induce apoptotic cell death. (A) Assessment of apoptosis via TUNEL assay. SK-MEL-2 and Malme-3M cells were treated with No Treatment, RNA Control (10 nM Scrambled DsiRNA), Drug Control (10 µM Enantiomer of ABT-737), 10 nM Mcl-1 DsiRNA, 10 µM ABT-737, 10 nM Scrambled DsiRNA and 10 µM ABT-737, or 10 nM Mcl-1 DsiRNA and 10 µM ABT-737. Cells were harvested for TUNEL 24 hours post-transfection. Induction of apoptosis is expressed as a fold increase over No Treatment. Data represent mean±SEM (n = 3). Statistical significance was determined using two-tailed, unpaired *t* tests. *P≤0.05 **P≤0.005, ***P≤0.0005. (B) Assessment of apoptosis via PARP cleavage. SK-MEL-2 and Malme-3M cells were treated with 10 nM Scrambled DsiRNA, 10 nM Mcl-1 DsiRNA and/or 10 µM ABT-737 in the combinations indicated for 24 hours.

We then confirmed that the observed decreases in viability were due to corresponding increases in apoptosis (Figure 4A). To quantify apoptosis, we measured DNA fragmentation using the TUNEL assay. In SK-MEL-2, which responds to both Mcl-1 DsiRNA and ABT-737 alone and in combination, there are statistically significant increases in apoptosis induction with all three treatments (Figure 4A). The marked increase in apoptosis with the combination treatment (Mcl-1 DsiRNA and ABT-737) confirms the synergistic effect on viability observed in Figure 3. In Malme-3M, which shows almost no change in viability with the single agents, there is also little change in apoptosis induction (Figure 4A). However, there is a significant increase in apoptosis with the combination treatment. Increases in apoptosis as assessed by TUNEL correspond to increased PARP cleavage by western immunoblot, demonstrating caspase-dependent death (Figure 4B).

### Combination treatment results in caspase-9-dependent cleavage of caspase-8, caspase-10 and Bid

**Figure 5:**
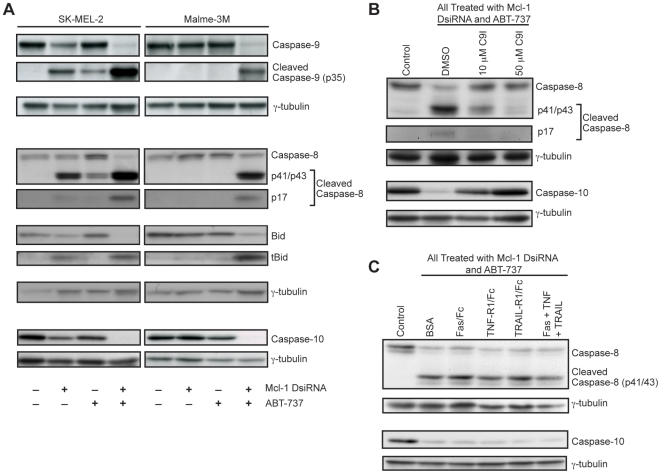
The combination of Mcl-1 knockdown and ABT-737 induces death receptor-independent cleavage of caspase-9, caspase-8, caspase-10 and Bid. (A) Western analysis showing caspase-9, -8, -10 and Bid levels with treatment. SK-MEL-2 and Malme-3M were treated with 10 nM Mcl-1 DsiRNA and 10 µM ABT-737, alone and in combination for 24 hours. (B) Western blots showing reduced caspase-8/-10 activation with inhibition of caspase-9. Lox IMVI cells were pretreated with 50 µM DMSO or the indicated doses of Z-LEHD-FMK (caspase-9 inhibitor, *C9I*) one hour prior to transfection. Cells were then treated with the combination of 10 nM Mcl-1 DsiRNA and 10 µM ABT-737. Control cells were pretreated with DMSO and transfected with Scrambled DsiRNA. (C) Western analysis showing that caspase-8/-10 activation is not affected by death receptor inhibitors. Lox IMVI cells were pre-treated with 1 µg/mL Fas/Fc, TNF-R1/Fc, TRAIL-R1/Fc or the combination of all three (all at 1 µg/mL) prior to transfection. Cells were then treated with 10 nM Mcl-1 DsiRNA and 10 µM ABT-737 for 24 hours.

A key event in the intrinsic pathway of apoptosis is cleavage of caspase-9 and formation of the apoptosome. A number of studies have shown that caspase-9 is cleaved following ABT-737 treatment [16], [26], [27]. We confirmed these results and further demonstrate that caspase-9 cleavage increases dramatically with the addition of Mcl-1 DsiRNA (Figure 5A).

Some studies have also observed increased caspase-8 cleavage with ABT-737 (single) treatment; however, the role of the extrinsic pathway in treatment response remains to be elucidated [16], [26], [27]. We observed caspase-8 cleavage following both Mcl-1 knockdown and ABT-737 treatment in all cell lines that respond to the single treatments (Figure 5A and data not shown). Moreover, caspase-8 cleavage dramatically increases with the combination treatment. A major target of active caspase-8 is the pro-apoptotic Bcl-2 protein Bid, which is cleaved to produce active, truncated Bid (tBid). Increased levels of cleaved caspase-8 are accompanied by increased levels of tBid (Figure 5A). Furthermore, the Mcl-1 DsiRNA and ABT-737 combination treatment significantly decreases levels of procaspase-10 (Figure 5A). Decreased procaspase-10 has previously been shown to correspond to caspase-10 cleavage and activation [28], [29], [30].

Caspase-8 and caspase-10 are initiator caspases of the death receptor pathway; however, there is some evidence that they can also be cleaved downstream of the mitochondria subsequent to caspase-9 activation [31]. We therefore assessed whether the caspase-8/-10 cleavage observed following combination treatment is dependent on caspase-9 activity. We demonstrate that inhibition of caspase-9 decreases cleavage of caspase-8 in a dose-dependent fashion (Figure 5B). At the 50 µM dose of the caspase-9 inhibitor, caspase-8 cleavage is almost completely abolished. The caspase-9 inhibitor also completely restores full-length caspase-10 (Figure 5B).

To assess the contribution of death receptor signaling to caspase-8/-10 activation, we used death receptor recombinant chimera (Fas/Fc, TNF-R1/Fc, and TRAIL-R1/Fc). These soluble receptors bind all corresponding ligand (both soluble and membrane-bound), thus preventing ligand from interacting with endogenous receptors and preventing death receptor-mediated apoptosis [32], [33]. We show that neither the individual recombinant chimera nor the combination of all three had any effect on caspase-8/-10 activation in response to Mcl-1 DsiRNA/ABT-737 treatment (Figure 5C).

### Response to combination treatment requires caspase activity but not death receptor signaling

**Figure 6:**
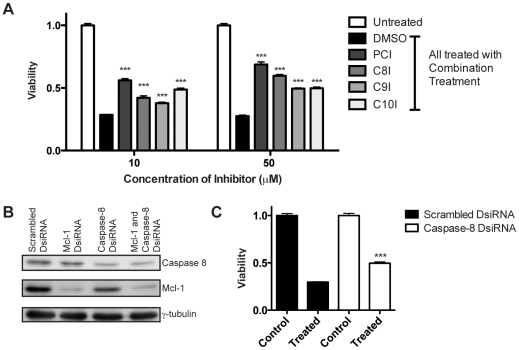
Inhibition of caspase-8, -9 and -10 reduces treatment efficacy. (A) Effects of Z-VAD-FMK (pan-caspase inhibitor, *PCI*) and Z-IETD-FMK (caspase-8 inhibitor, *C8I*), Z-LEHD-FMK (caspase-9 inhibitor, *C9I*) and Z-AEVD-FMK (caspase-10 inhibitor, *C10I*) on efficacy of the Mcl-1 DsiRNA and ABT-737 combination treatment. Lox IMVI cells were pretreated with DMSO or inhibitor 1 hour prior to transfection. Viability was assessed by MTT assay. Data represent mean±SEM (n = 4). Statistically significant differences between the DMSO treatment and the inhibitor treatments were determined using two-tailed, unpaired *t* tests. ***P≤0.0005. (B) Western immunoblot demonstrating knockdown of caspase-8. Lox IMVI cells were treated with Scrambled (40 nM), Mcl-1 (10 nM), caspase-8 (40 nM) or both Mcl-1 and caspase-8 DsiRNA for 24 hours. Immunoblotting was performed against both caspase-8 and Mcl-1 to demonstrate simultaneous knockdown. (C) Effects of caspase-8 knockdown on efficacy of the Mcl-1 DsiRNA and ABT-737 combination treatment. Cotransfection procedure as described in Materials and Methods. Viability was assessed by MTT assay. Data represent mean±SEM (n = 4). *P* value determined by two-tailed, unpaired *t* test ***P≤0.0005. Representative of three independent experiments.

To elucidate the pathways required for response to the combination treatment (Mcl-1 DsiRNA and ABT-737) we examined the effects of inhibitors of caspase-8 (Z-IETD-FMK), -9 (Z-LEHD-FMK) and -10 (Z-AEVD-FMK). The pan-caspase inhibitor Z-VAD-FMK is also shown for reference. All inhibitors significantly increase viability (and thus decrease treatment response) in cells treated with Mcl-1 DsiRNA and ABT-737 (Figure 6A).

To further confirm that caspase-8 plays a role in treatment response we also knocked down caspase-8 expression. Using DsiRNA, we were able to knockdown caspase-8 protein levels by ∼50% without interfering with Mcl-1 knockdown (Figure 6B). Pre-treatment with caspase-8 DsiRNA significantly increases viability in cells treated with the Mcl-1 DsiRNA/ABT-737 combination (P<0.0001; Figure 6C). Knockdown of caspase-8 was less effective in reducing treatment efficacy compared to the caspase-8 inhibitor, which may be due to incomplete knockdown. However, while the effect was not as strong as the peptide inhibitor, caspase-8 knockdown confirms that down-regulation of caspase-8 activity decreases efficacy of the combination treatment.

**Figure 7:**
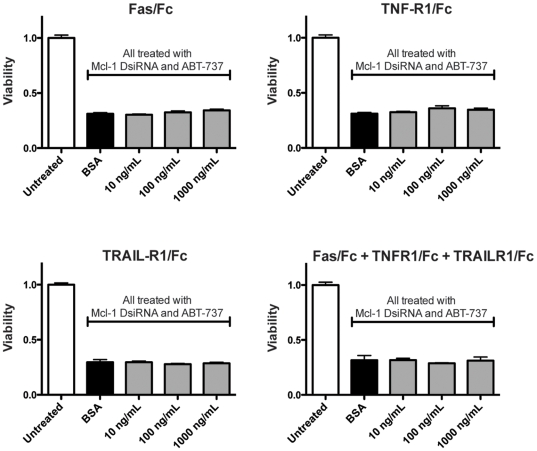
Inhibition of death receptor signaling does not affect treatment efficacy. Lox IMVI cells were pretreated with the recombinant chimera Fas/Fc, TNF-R1/Fc, TRAIL-R1/Fc or a BSA control (1 µg/mL) at the indicative doses prior to treatment with 10 nM DsiRNA, 10 µM ABT-737 for 24 hours. For the triple combination of inhibitors, the indicated dose represents the dose of each individual inhibitor. Viability was assessed by MTT assay. Data represent mean±SEM (n = 4).

To assess the requirement for death receptor signaling, we examined the effects of the recombinant chimera Fas/Fc, TNF-R1/Fc and TRAIL-R1/Fc on treatment response. Neither the individual chimera, nor the combination of all three, had any effect on viability of cells treated with the Mcl-1 DsiRNA and ABT-737 combination treatment (Figure 7).

### Levels of cleaved caspase-8 correlate with combination treatment response

Recent studies have demonstrated that expression levels of both pro- and anti-apoptotic Bcl-2 proteins affect ABT-737 sensitivity [34], [35]. However, to the authors' knowledge, no modifiers of the response to ABT-737 in combination with Mcl-1 knockdown have been identified. Given the dramatic caspase-8 and Bid cleavage observed following treatment with Mcl-1 DsiRNA and ABT-737 in melanoma, we investigated whether expression of either protein correlates with combination treatment response.

**Figure 8:**
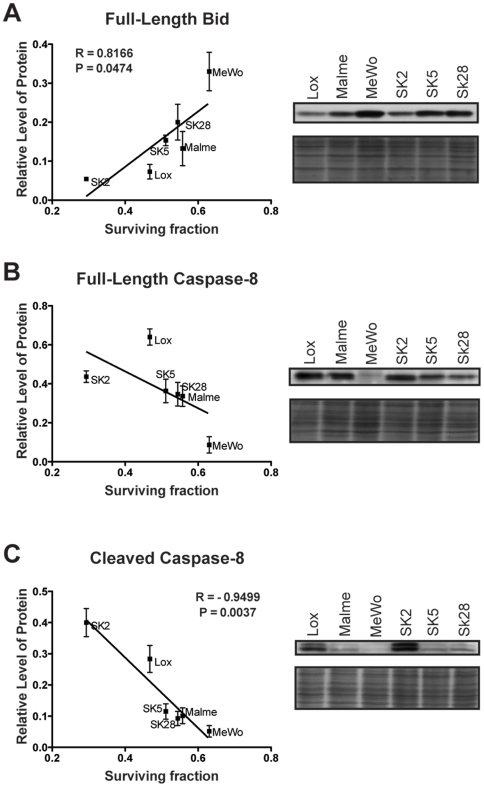
Levels of Bid and cleaved caspase-8 correlate with response to the combination treatment. *Right,* western analyses of endogenous levels of full-length Bid (A), full-length caspase-8 (B) and cleaved caspase-8 (C). Cells were harvested for protein at approximately equal confluency one day after splitting. *Left,* relative expression levels were quantified by densitometry, normalized to the amido black loading control, then plotted against the surviving fraction at 10 nM DsiRNA, 10 µM ABT-737 (expressed as a fraction of cells treated with Scrambled DsiRNA only). Data presented are the mean (±SEM) of three independent experiments. Correlation was assessed by Pearson correlation analysis: *R* (correlation coefficient) and *P* value are indicated on the graph. *Lox*, Lox IMVI; *Malme*, Malme-3M; *SK2*, SK-MEL-2; *SK5*, SK-MEL-5; *SK28*, SK-MEL-28.

As we were unable to calculate accurate EC_50_ values for all the cell lines (owing to the fact that some of the survival curves level off before reaching 50%) we calculated the surviving fraction of cells treated with 10 µM ABT-737, combined with our standard dose of 10 nM Mcl-1 DsiRNA. At this dose of the combination treatment, there is an almost two-fold difference in viability between the most responsive cell line (SK-MEL-2) and the least responsive (MeWo) (P<0.0001; Supplementary Figure S2). When the surviving fraction is plotted against basal expression levels of Bid and caspase-8, several trends are apparent. There is a statistically significant correlation between levels of Bid and survival, with the most responsive cell lines displaying lower levels of full-length Bid (Figure 8A). Cell lines with a strong response to treatment also have slightly higher levels of full-length procaspase-8 (Figure 8B). While we could not consistently detect tBid in untreated cells, we were able to detect and quantify the intermediate cleavage products of caspase-8 (p41/p43 isoforms). We demonstrated a strong, direct correlation between steady-state levels of cleaved caspase-8 and the surviving fraction (Figure 8C). As cleaved caspase-8 levels increase, there is an increase in treatment response. In contrast, there is no correlation between caspase-10 expression and treatment response (data not shown).

### Cell line differences are not due to known *CASP8* polymorphisms

In a recent report by Li *et al*., two putatively functional polymorphisms of *CASP8* were shown to contribute to melanoma susceptibility [36]. We therefore sequenced both the D302H (rs1045485:G>C) and −652 6N ins/del (rs3834129: −/CTTACT) polymorphisms in the six melanoma cell lines used in this study. None of the cell lines has the G to C transition to produce the D302H amino acid change. With regard to the −652 6N ins/del polymorphism: Malme-3M, MeWo, SK-MEL-2 and SK-MEL-5 are homozygous for the insertion allele, SK-MEL-28 is homozygous for the deletion and Lox IMVI is a heterozygote. The −652 6N deletion is thought to reduce expression of *CASP8* by eliminating an SP1 promoter binding site [37]. However, given that we observe highest levels of full-length caspase-8 in Lox IMVI (a heterozygote) and lowest levels in MeWo (an insertion homozygote), genotype at this locus likely does not play a major role in determining caspase-8 expression levels in the cell lines in this study.

## Discussion

Malignant melanoma is highly resistant to chemotherapeutic treatments largely due to an intrinsic resistance of the neoplastic melanocytes to undergo apoptosis. Our group was one of the first to describe increased expression of the anti-apoptotic protein Mcl-1 in melanoma [7]. In the present study, we have expanded these results using a tissue microarray. Mcl-1 expression increases with tumor depth and stage (Figure 1A,B) suggesting that Mcl-1 is a critical obstacle to apoptosis induction in melanoma.

However, we report that reduction of Mcl-1 expression alone is not enough to significantly reduce survival in multiple melanoma cell lines. We therefore combined Mcl-1 knockdown with an inhibitor of other anti-apoptotic Bcl-2 proteins, ABT-737, and demonstrate a synergistic effect of the combination treatment. We found that Mcl-1 DsiRNA enhances the effect of ABT-737 in all cell lines examined, resulting in significant decreases in overall viability (Figure 3) and increases in apoptosis induction (Figure 4). Thus, the combination therapy represents a promising new treatment strategy for malignant melanoma that calls for further investigation *in vivo*.

In initial mouse model studies, ABT-737 has been well-tolerated, with little effect on overall animal weight and no observed caspase activation in normal tissues such as liver, heart and intestine [17]. ABT-737 does appear to reduce platelet levels; however, the effect is reversible and platelet counts are rapidly restored post-treatment [38]. Future studies using mouse melanoma models will be required to assess both the anti-tumor activity and toxicity of our proposed combination therapy *in vivo*.

Many chemotherapeutic treatments act through activation of the death receptor pathway and subsequent cleavage of caspase-8 or -10 [39]. However, death receptor independent caspase-8/-10 activation has been reported in response to several anti-cancer agents including camptothecin, paclitaxel (Taxol) and etoposide [29], [40], [41]. In such instances, the active caspase is thought to function as part of a feedback loop to enhance the caspase cascade and amplify apoptotic signals. Loss or inhibition of caspase-8 or -10 significantly reduces the efficacy of these treatments. Thus death receptor-independent activation of caspase-8/-10 appears to be an important apoptotic mechanism for certain chemotherapeutic agents. Here we demonstrate death receptor-independent cleavage of caspase-8 and -10 as well as downstream target Bid following treatment with Mcl-1 DsiRNA and ABT-737 (Figure 5). Inhibition of either caspase-8 or caspase-10 (as well as caspase-8 knockdown) significantly reduces treatment efficacy suggesting that this downstream pathway may play an important role in the response of melanoma cells to the combination treatment (Figure 6).

By identifying the genetic factors involved in the induction of cell death following treatment, the long-term goal is to tailor therapy to individual tumor characteristics. We therefore sought to identify factors that modify sensitivity of melanoma cells to the combination treatment of Mcl-1 DsiRNA and ABT-737. We found a correlation between low levels of Bid in cell lines with greater treatment sensitivity (Figure 8). While this appears paradoxical, as Bid is pro-apoptotic, our data is concordant with a report on cervical cancer demonstrating a correlation between high Bid expression and poor radiotherapy outcome [42]. We propose that low levels of full-length Bid correspond to higher levels of cleaved Bid, although the cleavage product is difficult to detect owing to its short half-life. There are only slightly higher levels of full-length procaspase-8 in highly responsive cell lines; however, there is a strong, linear relationship between cleaved caspase-8 (p43/p41) and treatment response. Presumably, high levels of the intermediate cleavage products of caspase-8 correlate with high levels of the active protein (p17). Given that a caspase-8 dependent pathway appears to play an important role in treatment response, cell lines with high levels of active caspase-8 and Bid may therefore be better “primed” to die upon treatment with Mcl-1 DsiRNA/ABT-737. Thus, high levels of cleaved caspase-8 and, to a lesser extent, low levels of full-length Bid could potentially be used as predictors of response to ABT-737 and Mcl-1 knockdown.

There is currently no effective treatment for malignant melanoma. Here we present evidence that the combination of ABT-737 and Mcl-1 knockdown by DsiRNA efficiently induces cell death in multiple melanoma cell lines well over the effect seen with the individual treatments. Furthermore, our results indicate that a death receptor-independent pathway involving caspase-8, caspase-10 and Bid activation is important to combination treatment response. Further elucidation of the apoptotic pathways involved in treatment response will aid in the effective implementation of an ABT-737 and Mcl-1 knockdown-based therapy in malignant melanoma.

## Materials and Methods

### Ethics Statement

The use of human skin tissues in this study was approved by the medical ethics committee of the University of Alberta and was performed in accordance with the Declaration of Helsinki Principles. Only tissue that was initially taken for diagnostic purposes, and only secondarily used for research purposes, was used in this study. Because the samples were considered ‘biological samples normally discarded’ and were thus de-identified (ie no name, no reference to the hospital), the University of Alberta ethics board did not require specific patient consent.

### Antibodies, reagents and cell lines

Antibodies to Bid (#2003), total caspase-8 (#9746), cleaved caspase-8 (#9496), total caspase-9 (#9502), cleaved caspase-9 (35 kDa fragment; #9505), cleaved PARP (#9541), and full-length caspase-10 (9752) were purchased from Cell Signaling (Beverly, MA). Antibodies to Mcl-1 (M8434) and γ-tubulin (T6557) were purchased from Sigma (St. Louis, MO). Caspase inhibitors Z-IETD-FMK, Z-LEHD-FMK, and Z-VAD-FMK were obtained from Calbiochem, EMD Chemicals (San Diego, CA). Caspase-10 inhibitor Z-AEVD-FMK and recombinant chimera (Fas/Fc, TNF-R1/Fc, TRAIL-R1/Fc) were from R&D Systems (Minneapolis, MN). The recombinant chimera were dissolved in 0.1% BSA in PBS. ABT-737 and its enantiomer were generously supplied by Abbott Laboratories (Abbott Park, IL). Both compounds were dissolved in DMSO and stored at -20°C. Human melanoma cell lines were maintained in RPMI-1640 (Hyclone, Logan, UT) supplemented with 10% FBS (Hyclone). Human epidermal melanocytes were plated in Melanocyte Medium with melanocyte growth supplement, 0.5% FBS and 1% penicillin/streptomycin (all from ScienCell, Carlsbad, CA). After the second passage, melanocytes were maintained in RPMI-1640 with 10% FBS for at least 48 hours prior to harvest.

### Tissue Microarray (TMA)

Formalin-fixed, paraffin-embedded tissues from 28 human normal nevi, 49 primary melanomas and 32 metastatic melanomas were obtained from the Department of Laboratory Medicine and Pathology at the University of Alberta. The most representative tumor area was carefully selected and marked on the hematoxylin and eosin-stained slide. 0.6 mm-thick tissue cores were taken from each biopsy specimen. Multiple 4-µm sections were cut with a Leica microtome (Leica Microsystems Inc, Bannockburn, IL) and transferred to adhesive-coated slides.

### Immunohistochemistry of TMA

The TMA slides were dewaxed with xylene and rehydrated with graded alcohol washes. Antigen retrieval was performed by heating the samples at 95°C for 30 minutes in 10 mM sodium citrate (pH 6.0). Endogenous peroxidase activity was blocked with 3% hydrogen peroxide for 20 minutes. After blocking with universal blocking serum (DAKO Diagnostics, Mississauga, ON, Canada) for 30 minutes, the slides were incubated with anti-Mcl-1 antibody (1∶800) at 4°C overnight. The sections were incubated with biotin-labeled secondary antibody and streptavidin-peroxidase for 30 minutes each (DAKO Diagnostics). The samples were developed with 3,3′-diaminobenzidine substrate (Vector Laboratories, Burlington, ON, Canada) and counterstained with hematoxylin. Negative controls omitted Mcl-1 antibody during the primary antibody incubation.

### Evaluation of immunostaining

Mcl-1 staining in the TMA was scored by a pathologist and the total staining score was derived from the staining intensity multiplied by the percentage positive cells. Score for staining intensity: 0 = negative, 1 = weak staining, 2 = moderate staining, 3 = strong staining. Score for positive percentage: 1 = <20% positive, 2 = 20–39% positive, 3 = 40–59% positive, 4 = 60–79% positive, 5 = 80–100% positive.

### siRNA transfection

Cell lines were transfected with the indicated doses of Dicer-substrate siRNA (DsiRNA) directed against Mcl-1 (HSC.RNAI.N021960.2.3), caspase-8 (ACC NM_001228.4_3) or a universal negative control scrambled sequence (DS Scrambled Neg) (Integrated DNA Technologies, Coralville, IA) using Lipofectamine 2000 (Invitrogen, Carlsbad, CA) according to manufacturer's instructions. Where indicated, cells were treated with the appropriate dose of ABT-737 eight hours after DsiRNA transfection. To assess the effect of caspase-8 knockdown on survival, cells were first transfected with 40 nM caspase-8 or Scrambled DsiRNA in 60 mm plates, then split into 96 well plates 24 hours post-transfection. 24 hours later, cells were transfected with Mcl-1 or Scrambled DsiRNA and/or treated with ABT-737 as indicated.

### Viability and Apoptosis assays

Percent viability was assessed colorimetrically using MTT (3-(4,5-dimethyl-thiazol-2-yl)-2,5-diphenyltetrazolium bromide; Sigma). Twenty-four hours after transfection, plates were incubated with 0.5 mg/ml MTT for 6 hours. Following incubation, the formazan precipitate was solubilized with 24∶1 isopropanol:HCl and the absorbance was measured at 570 nm. Percent viability was calculated in comparison to control treatments. To measure apoptosis: 24 hours post-transfection, adherent and suspension cells were collected and fixed in 1% paraformaldehyde. Terminal deoxynucleotidyl dUTP nick end labeling (TUNEL) was performed using the Apo-BrdU *In Situ* DNA Fragmentation assay kit (Biovision, Mountain View, CA) according to manufacturer's instructions. Labeled cells were analyzed by fluorescence-activated cell sorter (FACS; BD Biosciences, San Jose, CA).

### Western blot analysis

Adherent and suspension cells were collected at indicated time points. Whole cell lysis, SDS-PAGE and western immunoblotting were performed as previously described [43]. Densitometry was performed using Quantity One software (Bio-Rad, Mississauga, ON, Canada). All blots were done in triplicate. In experiments comparing treatments within the same cell line, γ-tubulin is used as the loading control; however, as γ-tubulin levels can vary between cell lines, for experiments comparing different cell lines, a representative section of the amido black stain is shown.

### PCR and sequencing

DNA was extracted using the QIAquick DNA extraction columns (Qiagen, Valencia, CA). Previously described primers were used to amplify the −652 6N del polymorphism [37], and the D302H polymorphism [36]. Bands were purified using the QIAquick Gel Extraction Kit (Qiagen) and sequenced using the BigDye Terminator cycle sequencing kit (Applied Biosystems, Foster City, CA). Sequencing reactions were run on 3130×1 or 3100-Avant Applied Biosystems genetic analyzers.

### Statistical analyses

All statistical analyses were performed on GraphPad Prism version 5 for Macintosh (GraphPad Software, San Diego, CA). To assess the synergistic effect of the combination treatment, we used two-way ANOVA as previously described [25]. Briefly, overall viability by MTT assay (ie absorbance at 570 nm without normalization) of cells treated with the combination treatment (Mcl-1 DsiRNA 10 nM and ABT-737 10 µM) were compared against cells treated with either single treatment or cells treated with only a Scrambled DsiRNA control. The same data is shown in its normalized form in Figure 3. P-values less than 0.05 were considered statistically significant.

## Supporting Information

Figure S1 Effect of ABT-737 compared to drug enantiomer on cell viability in melanoma cell lines. Cells were treated with 0, 0.1, 1, 10, or 50 µM of ABT-737 or enantiomer. Each dose was increased by two orders of magnitude before the logarithm was taken to allow us to graph all doses as positive values. Viability was assessed at 24 hours via MTT assay. Curves were normalized to the 0 µM treatment. Data points represent mean±SEM (n = 4).
(1.42 MB TIF)

Figure S2 Survival at 10 nM DsiRNA, 10 µM ABT-737 in 6 melanoma cell lines. Survival is expressed as a fraction of cells treated with Scrambled DsiRNA only (10 nM). Data represent mean±SEM (n = 4). The statistical significance of the difference between the least and most responsive cell lines (MeWo and SK-MEL-2, respectively) was determined by a two-tailed, unpaired t test.
(1.11 MB TIF)
