# Supplementary figures and images for: RNA Silencing of Mcl-1 Enhances ABT-737-Mediated Apoptosis in Melanoma: Role for a Caspase-8-Dependent Pathway
Source: PLoS One. 2009 Aug 17;4(8):e6651. doi: 10.1371/journal.pone.0006651 (PMC2722728; doi:10.1371/journal.pone.0006651)

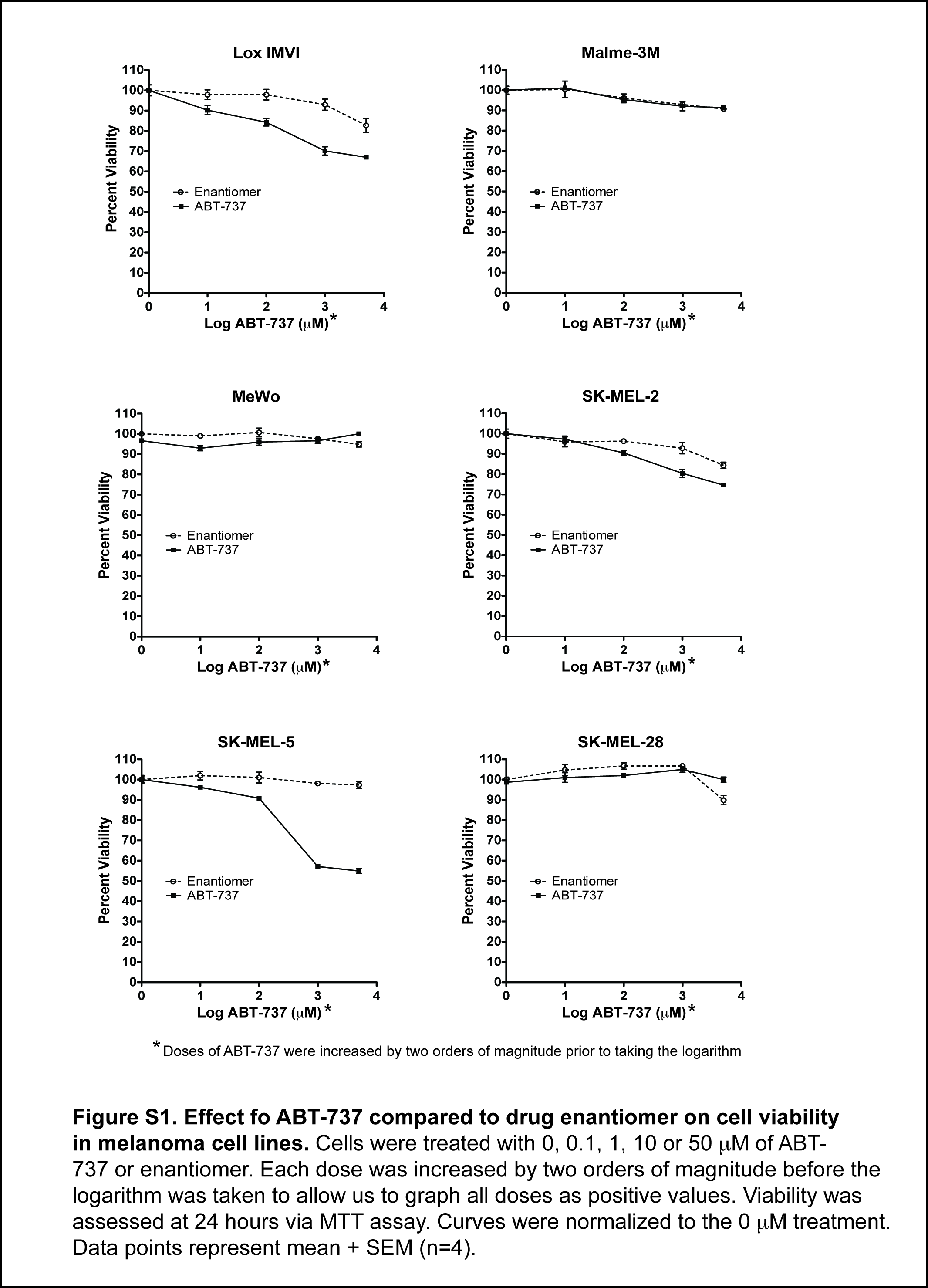

Supplement: Figure S1 — Effect of ABT-737 compared to drug enantiomer on cell viability in melanoma cell lines. Cells were treated with 0, 0.1, 1, 10, or 50 µM of ABT-737 or enantiomer. Each dose was increased by two orders of magnitude before the logarithm was taken to allow us to graph all doses as positive values. Viability was assessed at 24 hours via MTT assay. Curves were normalized to the 0 µM treatment. Data points represent mean±SEM (n = 4). (1.42 MB TIF) [file pone.0006651.s001.tif]

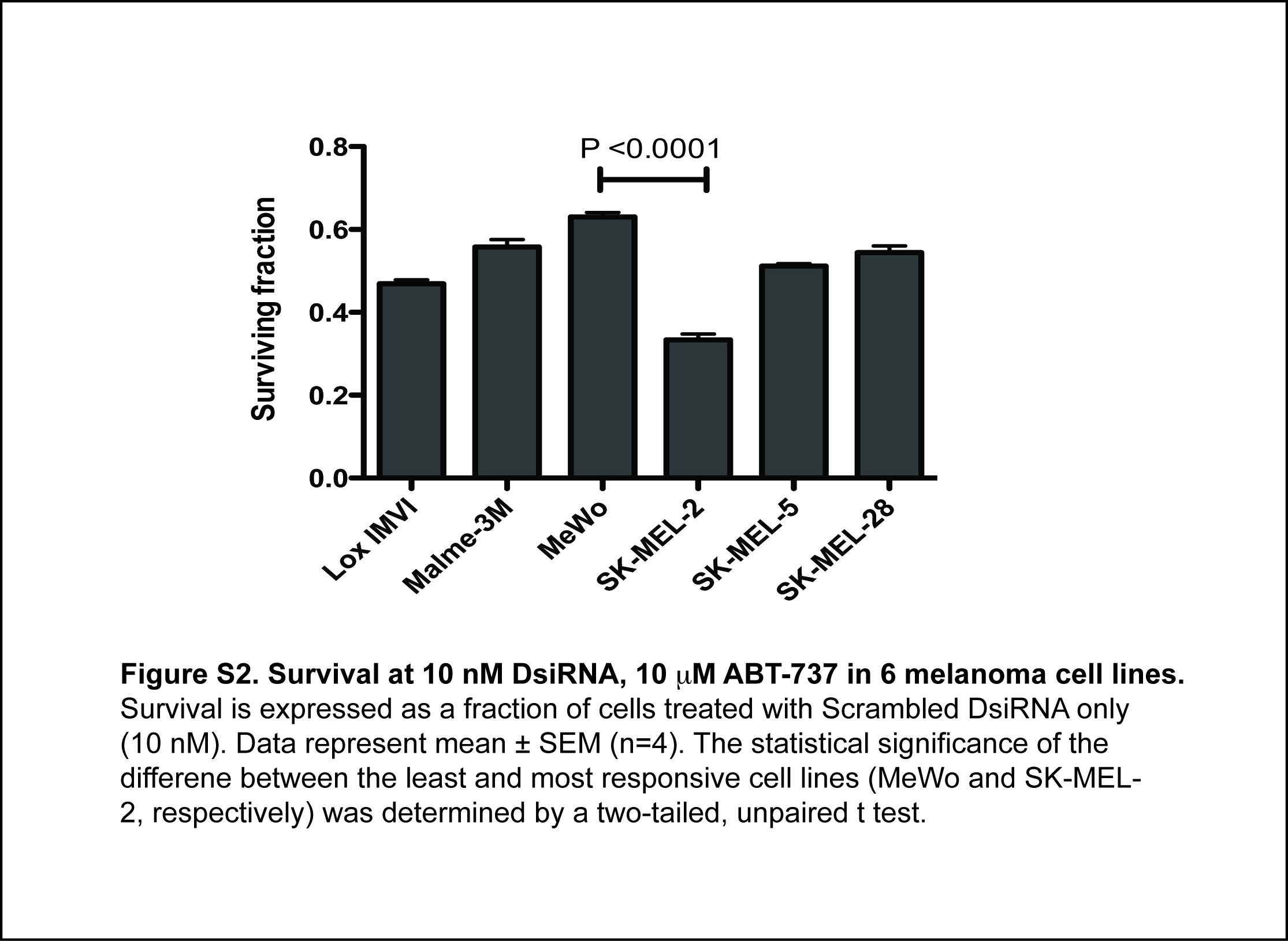

Supplement: Figure S2 — Survival at 10 nM DsiRNA, 10 µM ABT-737 in 6 melanoma cell lines. Survival is expressed as a fraction of cells treated with Scrambled DsiRNA only (10 nM). Data represent mean±SEM (n = 4). The statistical significance of the difference between the least and most responsive cell lines (MeWo and SK-MEL-2, respectively) was determined by a two-tailed, unpaired t test. (1.11 MB TIF) [file pone.0006651.s002.tif]
